# Supplementary material for: Nypa fruticans wurmb (Mangrove Palm) Fruit Kernel: Effect on physicochemical, sensory evaluation and shelf-life of wheat biscuits
Source: PLoS One. 2026 Feb 18;21(2):e0342665. doi: 10.1371/journal.pone.0342665 (PMC12915934; doi:10.1371/journal.pone.0342665)

PLOS ONE

Nypa fruticans (Mangrove Palm) Fruit Kernel: A Novel Ingredient for Nutrient-Dense, Low-Fat Biscuits

--Manuscript Draft--

**Manuscript Number:**	PONE-D-25-57201
**Article Type:**	Research Article
**Full Title:**	Nypa fruticans (Mangrove Palm) Fruit Kernel: A Novel Ingredient for Nutrient-Dense, Low-Fat Biscuits
**Short Title:**	Production of Biscuits
**Corresponding Author:**	Md. Ripaj Uddin, MS<br>Bangladesh Council of Scientific and Industrial Research<br>Dhaka, Dhaka BANGLADESH
**Keywords:**	Nypa fruticans kernel Flour; Gluten-Free Biscuits; Nutritional improvement; Sensory acceptability; Sustainable food ingredient and Shelf-life
**Abstract:**	The global biscuit market is evolving, with increasing consumer demand for healthier, functional, and gluten-free alternatives to traditional wheat-based products. This necessitates the exploration of novel, sustainable flour sources. The Nipa palm (Nypa fruticans), a prolific mangrove plant, produces an underutilized kernel (NFK) that holds potential as a nutritious, non-conventional flour. While studies have explored various alternative flours, the application of Nipa palm kernel flour in biscuit production remains largely unexplored. This research introduces NFK as a novel, gluten-free functional ingredient, aiming to valorize an underutilized resource and develop a healthier snack option. Mature Nipa fruits were collected from the Sundarbans, Bangladesh. Kernels were extracted, blanched, dried, and milled into flour. Biscuits were formulated by substituting refined wheat flour with 0% (control), 10%, 20%, and 30% NFK. The physical, nutritional, sensory, and shelf-life properties of the biscuits were comprehensively analyzed using standard methods. NFK incorporation significantly improved the biscuits' nutritional profile. The 30% NFK biscuit exhibited a 38% reduction in fat, a seven-fold increase in crude fibre (from 0.19% to 1.35%), and a substantial boost in essential minerals, with iron content more than doubling to 53.3 mg/kg. Physically, NFK biscuits had a higher spread ratio, softer texture, and a desirable darker color. Crucially, all NFK-enriched biscuits received high sensory acceptability scores (≥7.85 on a 9-point scale), with no significant difference in overall liking compared to the control. Microbiological analysis confirmed product safety and a shelf life of over 12 months. The study was conducted at a laboratory scale. Further research should investigate large-scale production feasibility, in-vivo bioavailability of the enhanced minerals, and the precise characterization of NFK's bioactive compounds. Nipa palm kernel flour is a highly promising functional ingredient for developing nutritious, sensorily-accepted, and gluten-free biscuits, promoting the sustainable use of mangrove resources and contributing to diversified healthy food choices.
**Order of Authors:**	Md. Ripaj Uddin, MS
	Md. Salim Khan
	Md. Khairul Islam
	Muhammad Abdullah Al Mansur
	Md. Selim Reza
	Md Hamedul Islam
	Mehedi Hasan
	Sharmin Ahmed
	Sirajum Monira
	Abubakr M. Idris

Powered by Editorial Manager® and ProduXion Manager® from Aries Systems Corporation

Additional Information:	
Question	Response
**Financial Disclosure**<br><br>Enter a financial disclosure statement that describes the sources of funding for the work included in this submission. Review the submission guidelines for detailed requirements. View published research articles from *PLOS ONE* for specific examples.<br><br>This statement is required for submission and **will appear in the published article** if the submission is accepted. Please make sure it is accurate.<br><br>**Funded studies**<br>Enter a statement with the following details:<br>• Initials of the authors who received each award<br>• Grant numbers awarded to each author<br>• The full name of each funder<br>• URL of each funder website<br>• Did the sponsors or funders play any role in the study design, data collection and analysis, decision to publish, or preparation of the manuscript?<br><br>Did you receive funding for this work?	The author(s) received no specific funding for this work.
**Competing Interests**<br><br>Use the instructions below to enter a competing interest statement for this submission. On behalf of all authors, disclose any competing interests that could be perceived to bias this work—acknowledging all financial support and any other relevant financial or non-financial competing interests.<br><br>This statement is required for submission and **will appear in the published article** if the submission is accepted. Please make sure it is accurate and that any funding sources listed in your Funding Information	The authors have declared that no competing interests exist

later in the submission form are also declared in your Financial Disclosure statement.<br><br>View published research articles from *PLOS ONE* for specific examples.<br><br>**NO authors have competing interests**<br><br>Enter: *The authors have declared that no competing interests exist.*<br><br>**Authors with competing interests**<br><br>Enter competing interest details beginning with this statement:<br><br>*I have read the journal's policy and the authors of this manuscript have the following competing interests: [insert competing interests here]*<br><br>* typeset	
**Ethics Statement**<br><br>Enter an ethics statement for this submission. This statement is required if the study involved:<br><br>• Human participants<br>• Human specimens or tissue<br>• Vertebrate animals or cephalopods<br>• Vertebrate embryos or tissues<br>• Field research<br><br>Write "N/A" if the submission does not require an ethics statement.<br><br>General guidance is provided below. Consult the submission guidelines for detailed instructions. **Make sure that all information entered here is included in the Methods section of the manuscript.**	(involving human participants)

**Format for specific study types**<br><br>**Human Subject Research (involving human participants and/or tissue)**<br>• Give the name of the institutional review board or ethics committee that approved the study<br>• Include the approval number and/or a statement indicating approval of this research<br>• Indicate the form of consent obtained (written/oral) or the reason that consent was not obtained (e.g. the data were analyzed anonymously)<br><br>**Animal Research (involving vertebrate animals, embryos or tissues)**<br>• Provide the name of the Institutional Animal Care and Use Committee (IACUC) or other relevant ethics board that reviewed the study protocol, and indicate whether they approved this research or granted a formal waiver of ethical approval<br>• Include an approval number if one was obtained<br>• If the study involved *non-human primates*, add *additional details* about animal welfare and steps taken to ameliorate suffering<br>• If anesthesia, euthanasia, or any kind of animal sacrifice is part of the study, include briefly which substances and/or methods were applied<br><br>**Field Research**<br><br>Include the following details if this study involves the collection of plant, animal, or other materials from a natural setting:<br>• Field permit number<br>• Name of the institution or relevant body that granted permission	
**Data Availability**<br><br>Authors are required to make all data underlying the findings described fully available, without restriction, and from the time of publication. PLOS allows rare exceptions to address legal and ethical concerns. See the PLOS Data Policy and FAQ for detailed information.	Yes - all data are fully available without restriction

A Data Availability Statement describing where the data can be found is required at submission. Your answers to this question constitute the Data Availability Statement and **will be published in the article**, if accepted.<br><br>**Important:** Stating 'data available on request from the author' is not sufficient. If your data are only available upon request, select 'No' for the first question and explain your exceptional situation in the text box.<br><br>Do the authors confirm that all data underlying the findings described in their manuscript are fully available without restriction?	
**Describe where the data may be found in full sentences. If you are copying our sample text, replace any instances of XXX with the appropriate details.**<br><br>• If the data are **held or will be held in a public repository**, include URLs, accession numbers or DOIs. If this information will only be available after acceptance, indicate this by ticking the box below. For example: *All XXX files are available from the XXX database (accession number(s) XXX, XXX.*).<br>• If the data are all contained **within the manuscript and/or Supporting Information files**, enter the following: *All relevant data are within the manuscript and its Supporting Information files.*<br>• If neither of these applies but you are able to provide **details of access elsewhere**, with or without limitations, please do so. For example:<br><br>*Data cannot be shared publicly because of [XXX]. Data are available from the XXX Institutional Data Access / Ethics Committee (contact via XXX) for researchers who meet the criteria for access to confidential data.*<br><br>*The data underlying the results presented in the study are available from (include the name of the third party*	All data are available in the manuscript.

and contact information or URL).<br>• This text is appropriate if the data are owned by a third party and authors do not have permission to share the data.<br><br>* typeset	
Additional data availability information:	

Powered by Editorial Manager® and ProduXion Manager® from Aries Systems Corporation

Manuscript Click here to access/download;Manuscript;Manuscript.docx

Nypa fruticans (Mangrove Palm) Fruit Kernel: A Novel Ingredient for Nutrient-Dense, Low-Fat Biscuits

Md. Ripaj Uddin[1*], Md. Salim Khan[1], Md. Khairul Islam[4], Muhammad Abdullah Al Mansur[1], Md. Selim Reza[3], Md Hamedul Islam[4], Mehedi Hasan[1], Sharmin Ahmed[1], Sirajum Monira[5], Abubakr M. idris[6,7]

[1]*Institute of National Analytical Research and Service (INARS), BCSIR, Dhaka 1205, Bangladesh.*

[2]*Institute of Mining, Mineralogy and Metallurgy (IMMM), Bangladesh Council of Scientific and Industrial Research (BCSIR), Joypurhat-5900.*

[3]*Department of Biochemistry & Molecular Biology, Jahangirnagar University, Savar, Dhaka-1342 Bangladesh.*

[4]*Departrnent of Pharmacy, Dhaka International University, Satarkul, Bradda, Dhaka, Bangladesh.*

[5]*Geological Survey of Bangladesh, Ministry of power, energy & mineral resources, Dhaka-1000.*

[6]*Department of Chemistry, King Khalid University, College of Science, P.O. Box 9004, Abha 62529, Saudi Arabia*

[7]*Research Center for Advanced Materials Science (RCAMS), King Khalid University, Abha 62529, Saudi Arabia.*

[*]Corresponding author: E-mail address: md.ripajuddin@gmail.com (M.R.U)

Abstract: The global biscuit market is evolving, with increasing consumer demand for healthier, functional, and gluten-free alternatives to traditional wheat-based products. This necessitates the exploration of novel, sustainable flour sources. The Nipa palm (*Nypa fruticans*), a prolific mangrove plant, produces an underutilized kernel (NFK) that holds potential as a nutritious, non-conventional flour. While studies have explored various alternative flours, the application of Nipa palm kernel flour in biscuit production remains largely unexplored. This research introduces NFK as a novel, gluten-free functional ingredient, aiming to valorize an underutilized resource and develop a healthier snack option. Mature Nipa fruits were collected from the Sundarbans, Bangladesh. Kernels were extracted, blanched, dried, and milled into flour. Biscuits were formulated by substituting refined wheat flour with 0% (control), 10%, 20%, and 30% NFK. The physical, nutritional, sensory, and shelf-life properties of the biscuits were comprehensively analyzed using standard methods. NFK incorporation significantly improved the biscuits' nutritional profile. The 30% NFK biscuit exhibited a 38% reduction in fat, a seven-fold increase in crude fibre (from 0.19% to 1.35%), and a substantial boost in essential minerals, with iron content more than doubling to 53.3 mg/kg. Physically, NFK biscuits had a higher spread ratio, softer texture, and a desirable darker color. Crucially, all NFK-enriched biscuits received high sensory acceptability scores (≥7.85 on a 9-point scale), with no significant difference in overall liking compared to the control. Microbiological analysis confirmed product safety and a shelf life

of over 12 months. The study was conducted at a laboratory scale. Further research should investigate large-scale production feasibility, in-vivo bioavailability of the enhanced minerals, and the precise characterization of NFK's bioactive compounds. Nipa palm kernel flour is a highly promising functional ingredient for developing nutritious, sensorily-accepted, and gluten-free biscuits, promoting the sustainable use of mangrove resources and contributing to diversified healthy food choices.

Key Words: *Nypa fruticans* kernel Flour, Gluten-Free Biscuits, Nutritional improvement, Sensory acceptability, Sustainable food ingredient and Shelf-life.

Introduction

Biscuits are well known baked foods with low moisture content and have long shelf life, which are considered as a convenient, inexpensive snack among all the age groups across the world [1]. With the increased consumption of biscuit products, there is also an increasing need for their dietary functionality [2]. Biscuits contain various ingredients such as flour, sugar, milk, eggs, fats or shortening and flavoring ingredients [1]. The flour-based component of biscuits used as raw material and largely are wheat based and have a high gluten content is (approximately 12–14 %) [3]. Nonetheless, due to increasing consumer preference for natural, healthy and functional foods and a high level of competition in the market, food companies are beginning to replace traditional flours with new ones including corn, flax seed, potato flour and defatted rice bran or other non-gluten grains like beans in developing formulations [4]. The Nipa palm (*Nypa fruticans* Wurmb) is a mangrove palm, and the only member of the Genus :Nypa. Known as “golpata” in Bangladesh, this plant is common throughout the mangrove forests of Asia, Oceania and east Africa [5, 6].

In Bangladesh, it exists in the coastal and tidal area especially in the Sundarbans [7]. Although the plant, *N. fruticans* is available commercially in the wild, rural farmers residing in the southwestern districts of Patuakhali, Bagerhat, Khulna and Satkhira grow this plant as a cash crop in agricultural fields for domestic purpose. Its tough leaves are an important commodity, often employed for thatching houses in these areas [8]. Besides its construction uses, the inflorescences of *N. fruticans* can be tapped to collect a sap that may be fermented to vinegar and alcohol or boiled for brown sugar [9]. In addition, *N. fruticans* is highly productive in fruit production and can bear fruits for several times a year. Fruit bundles have 30–50 individual fruits, and each ripe fruit consists of a

brownish epicarp, fibrous mesocarp, thin endocarp and central whitely developed kernel [10]. This palm kernel, freshly "white" from mature fruit of *N. fruticans* is potential to be converted to flour. *N. fruticans* fruit kernel meal is a rich ingredient in terms of nutritional composition, presenting high carbohydrates (74.57 to 87.56%) and dietary fibers (0.18–17.68%), but low amounts of lipids (0,40–12,25%) and proteins (2.66–8.17%) [11; 12]. The kernel is also a good source of vitamins A (2.57 mg/100g) and C (20.36 mg/ 100g) as well as essential minerals such as magnesium,, potassium, calcium, sodium, manganese iron and copper. Despite the existence of anti-nutritive factors such as hydrogen cyanide, tannins, phytic acid and oxalates in the ripe fruit kernels of *N. fruticans* cultivars have concentrations that do not exceed lethal poisoning for humans (2.5 g/100g) [11]. Consequently, flour of *N.fruticans* fruit kernel may play a role as a nutritive factor in food products. In the context of biscuit sector competition and market pressure to gluten-free product, *N. fruticans* fruit kernel flour can be considered as a potential substitute for wheat flour [13]. Introduction of this ruin-free flour into biscuit manufacturing would not only address the requirement of health-oriented end-consumers with special dietary needs but using waste nutrition product it will also lead to sustainability and promote new product development [14]. The description of NFK valorization still can add to national economic development and infrastructure integrity and food security as well as job oppurtunities.

The objective of the present study is to investigate the nutritional composition and consumer acceptability of the biscuits with N-kind flour from *Nypa fruticans* (N), schard kernel and further to assess changes in their nutrient content after baking. By producing biscuits with NFK as functional ingredient, a high-quality gluten-free and healthy snack item can be introduced while enhancing the sustainable utilization of an undervalued resource.

2. Methods and Materials

2.1.1. Source of *Nypa fruticans* fruit and Ethical Approval.

The mature fruits of *Nypa fruticans* were collected from the estuarine mangroves of the Sundarbans in Bangladesh (about 21.5ºN - 22.5ºN, 89.0ºE – 90.0ºE) during the fruiting season period (02 January, 2024 to 30 March, 2024). Fruits were chosen for the uniform brown colored exocarps, comparable size and not involving physically damaged or microbial infested. A voucher

specimen was identified (Voucher Specimen No: DACB 12345). It fits within the context of the previous studies which have been undertaken on underutilised mangrove resources [15, 16].

This research was approved ethically by the Committee of Clinical Pharmacy & Pharmacology, Faculty of Pharmacy, Dhaka International University (Ref No: CPP/DIU/EC/22).

2.1.2. Kernel Extraction and Flour Preparation

NFK flour Kernels (Fig. 1) from *Nypa fruticans* (NF) were milled according to the modified method for palm kernels [17, 18]. The fibrous epicarp was removed manually by steel knife and the nuts were cracked by opening it to remove the kernel. The kernels were washed with drinking water before rinsing twice with 0.5% solution of sodium metabisulfite to avoid enzymatic browning. Thereafter, samples were blanched at 80 °C for 5 min to inactivate polyphenol oxidase and lipase enzymes [19]. The blanched kernels were oven dried at 60 °C for 48 hours to retain heat-labile nutrients [20] and ground with hammer mill (Retsch SK 100). The dried minced barley was passed through an 80-mesh screen (180 µm) in order to ensure that the particle size was consistent with that of white wheat flour [21]. Finally, the NFK flour was sealed in nitrogen-flushed high-density polyethylene (HDPE) bags with oxygen absorbers and kept in a desiccator 25 ± 2 °C to avoid oxidative deterioration until ready for analysis [22].

Fig. 1. Making biscuits utilizing NFK Flour.

2.2 Proximate Composition Analysis of NFK Flour

The proximate composition of the samples including moisture, ash, crude protein, crude fat, crude fiber, and total carbohydrates was determined using established official methods. Moisture content was determined gravimetrically according to AOAC 934.01. Briefly, a known weight of the sample was dried to a constant weight in a hot-air oven (Memmert GmbH + Co. KG, Schwabach, Germany) at 105°C. The moisture content was calculated as the percentage of weight loss due to water evaporation using the equation No. 1 [23]:

$$\text{Moisture (\%)} = \frac{W - W_1}{W} \times 100 \quad \ldots\ldots (1)$$

Where, W= Weight of fresh sample (g), and W1= Weight of dried sample (g).

Ash (total mineral matter) was analyzed via the direct method outlined in AOAC 942.05. The gross energy value was subsequently calculated from these results. A pre-weighed sample was

incinerated in a muffle furnace (Nabertherm GmbH, Lilienthal, Germany) at 550°C for 5 hours until a constant white ash was obtained. The ash content was expressed as a percentage of the original sample weight calculated as equation No. 2 [23]:

$$\text{Ash (\%)} = \frac{W_1}{W} \times 100 \quad (2)$$

Where, W= Weight of sample (g) and W1= Weight of ash (g).

Crude protein content was estimated by determining the total nitrogen content using the Kjeldahl method as per AOAC 984.13 (A-D). The process involved digestion of the sample with concentrated sulfuric acid in the presence of a catalyst to convert organic nitrogen to ammonium sulfate, followed by distillation and titration of the liberated ammonia with a standardized acid. The nitrogen content was converted to crude protein using a specific conversion factor as shown in the equation No. 3 [23, 26]:

$$\text{Crude Protein (\%)} = \frac{(B-S)\times N\times 1.007}{W} \times F \quad (3)$$

where, B= mL of acid blank, S= mL of acid sample, N= Normality of acid, W= Weight of sample (g) and F=5.70 (nitrogen-to-protein conversion factor) as recommended by the codex standards.

Crude fat content was determined by continuous solvent extraction using a Soxhlet apparatus following the AOAC 920.39 (A) method for crude fat. A known weight of the dried sample was subjected to extraction with petroleum ether (boiling point range 40-60°C) for 6 hours. The solvent was then evaporated and recovered, and the fat content was determined gravimetrically by weighing the flask before and after extraction and expressed as a percentage equation No. 4 [23]:

$$\text{Crude Fat (\%)} = \frac{W_1}{W} \times 100 \quad (4)$$

Where, W1= Weight of fat (g) and W= Weight of sample (g)

Crude fiber content was analyzed according to the AOAC 962.09 method. The defatted sample was subjected to sequential digestion with 1.25% H_2SO_4 and 1.25% NaOH solutions under controlled conditions to remove soluble carbohydrates and proteins. The remaining insoluble organic residue was filtered, dried, and weighed, then ashed in a muffle furnace at 550°C. The loss in weight upon ashing represents the crude fiber, calculated as equation No. 5 [24]:

$$\text{Crude Fiber (\%)} = \frac{W_1 - W_2}{W} \times 100 \quad (5)$$

Where, W1=Weight of residue after digestion (g), W2=Weight of ash (g) and W=Weight of sample (g)

Total carbohydrate content was calculated by difference, a standard method endorsed by numerous authorities including the Food and Agriculture Organization (FAO) [27], as it accounts for all other unmeasured components. The percentage of total carbohydrates was obtained by subtracting the sum of the percentages of moisture, ash, crude protein, and crude fat from 100 equation No. 6 [25]:

Total Carbohydrates (%) $= 100 - (X + Y + A + B)$ …………………………….. (6)

Where, X= Moisture %, Y= Ash %, A= Crude Protein % and B=

Finally, the energy content was calculated from the proximate composition using the modified Atwater general factors as described by Merrill and Watt which assign 4 kcal/g for proteins, 9 kcal/g for fats, and 4 kcal/g for carbohydrates. The gross energy was calculated using the formula No 7 [25, 27]:

Energy (kcal/100g) $= 4 \times A + 9 \times B + 4 \times C$……………………………….(7)

While, A=Crude Protein (g), B=Crude Fat (g) and C=Total Carbohydrates (g)

2.3 Mineral and Toxic Metal Analysis with Quality Control

The mineral and toxic metal profile of the *Nypa fruticans* kernel powder was determined using atomic absorption spectrophotometry (AAS) following a rigorous digestion and analytical protocol to ensure accuracy and precision. All procedures adhered to standard methods from the American Public Health Association (APHA) [28, 29] and the quality assurance system of ISO/IEC 17025:2017 [30]. All glassware and volumetric equipment were meticulously cleaned by soaking overnight in a 2% (v/v) nitric acid (HNO_3) solution prepared with deionized water (18.2 MΩ·cm resistivity, 0.2 µS/cm conductivity) and thoroughly rinsed prior to use to prevent any cross-contamination [31-33].

A precisely weighed 2-gram sample of the homogenized kernel powder was subjected to a wet digestion process in a calibrated fume hood. The sample was placed in a high-quality borosilicate glass beaker and digested with 20 mL of 70% analytical grade HNO_3 (Sigma-Aldrich, Germany) on a hot plate at 90°C for approximately two hours until a clear digestate was obtained. The

digested sample was then cooled, filtered through a Whatman No. 42 ashless filter paper to remove any particulate matter, and the filtrate was quantitatively transferred into a 100 mL volumetric flask where the volume was made up to the mark with deionized water. For the determination of toxic metals at very low concentrations, a more sensitive graphite furnace AAS (GF-AAS, Varian AA240Z with GTA 120) was employed for the analysis of Cr, Cd, As, and Pb. The analysis of essential minerals (Fe, Mn, Cu, Zn, Mg, Ca) was conducted using flame atomic absorption spectroscopy (FAAS; Varian SpectraAA 240FS/220). Concentrations of the major cations (Na and K) were determined by a calibrated flame photometer (Jenway PFP7, UK).

The entire analytical procedure followed a rigorous Quality Assurance and Quality Control (QA/QC) plan. The method was validated and calibrations were carried out using certified reference materials (CRMs) and high-purity reagents (Scharlau, Sigma-Aldrich). Standards Stock standard solutions (1000 mg/L) of each element were appropriately diluted to give calibration curves. Procedure reliability was ascertained by spiking experiments, duplicate analyses of samples, independent standard checks and the analysis of procedural blanks in each batch. Data quality and precision were controlled through triplicate analysis of a NIST-traceable CRM in combination with the use of a control chart (99% confidence limit) to monitor results. The method detection limit (MDL), calculated as three times the standard deviation of the blank signal, was determined to be 0.005 mg/L for Cu, 0.01 mg/L for Cr, 0.001 mg/L for Pb, 0.05 mg/L for Cd, 0.1 mg/L for Mn, and 0.07 mg/L for Zn. The final concentration of each element in the sample was calculated after blank subtraction using the formula No 8:

$$\text{Element Concentration (mg/kg)} = \frac{(\mathrm{C} \times \mathrm{V} \times \mathrm{D})}{M} \quad (8)$$

where C is the concentration of the element in the digested solution (mg/L) obtained from the calibration curve, V is the final volume of the digest (L), D is any additional dilution factor, and M is the mass of the original sample (kg).

2.4 Biscuit Formulation and Preparation

Biscuits were formulated by partially substituting wheat flour with NFK powder to create nutrient-rich, low-fat products. A control biscuit (0% substitution) and several experimental biscuits with varying levels of NFK substitution (e.g., 10%, 20%, 30%) were developed based on a standard creamery-style short dough biscuit formula, adapted from methods commonly used in cereal

science research [34, 35] (Manley, 2011; Pareyt & Delcour, 2008). The control formulation consisted of wheat flour (1000 g), powdered sugar (250 g), fresh whole eggs (4 units, approximately 200 g), sodium chloride (5.0 g), sodium bicarbonate (2.0 g) as a leavening agent, dried yeast (4.0 g) for additional aeration, a reduced amount of shortening (30 g) to align with the low-fat objective, and vanilla essence (6 g) for flavour. The added water for dough formation was standardized to 500 ± 20 Brabender Units (BU, arbitrary units) across all batches and assessed empirically. The dough, which was creamed in order to aerate it uniformly and develop the crumb structure, consisted of the following constituents:

Briefly, shortening and sugar were creamed in a planetary mixer (KitchenAid 5K5SS, USA) with a flat beater at medium speed for 5 min. Then the eggs and vanilla extract were added gradually with continued mixing until it became a pale, airy emulsion. In a separate bowl, premixed wholewheatflour, NFK powder if used in the recipe, soda and yeast ascorbic acid were made up. This dry blend was added to the creamed portion and was combined at low speed minimum mixing to avoid overdevelopment of gluten structure. Water was added gradually until a stiff, muddy dough formed. Finally, the dough was rested for 10 min at 25°C to achieve complete hydration and gluten relaxation.

The relaxed dough was sheeted to 6.0 mm thickness by means of a small scale sheeter (Rondo GmbH, Switzerland) and cut into round sheets of 50 cm in diameter. The biscuits were transferred to a greased aluminium baking tray and baked in pre-heated forced convection oven (Model UNE400, Memmert GmbH + Co. KG, Germany) at 180°C for 12-15 minutes until light golden brown colour was obtained. The biscuits were then cooled at room temperature on wire racks for 1 h (to ensure that moisture had a chance to redistribute and the structure to develop).

The cooled biscuits were packaged in metallized polyester pouches with high oxygen and moisture barrier properties and heat-sealed. They were then stored at 20°C in a desiccator containing silica gel to retard moisture uptake and maintain stability until analysis. Baking loss was determined gravimetrically as the percentage of weight lost during baking due to water evaporation and the loss of volatile components, calculated using Equation (9) [36-38]:

$$\text{Baking Loss (\%)} = \frac{W_1 - W_2}{W} \times 100 \quad \text{(9)}$$

Where W is Weight of dough before baking (g), W_1 is Weight of dough before baking (g) and W_2 is Weight of biscuit after baking (g).

2.5 Analysis of Physical and Chemical Properties of Biscuits

Physical properties of the biscuits produced were investigated after 24 hours at room temperature and controlled humidity. The diameter and the thickness of ten randomly taken biscuits from each batch were measured using a digital vernier caliper (Mitutoyo, Corp., Japan) at the accuracy of 0.01 mm. Weight of individual biscuits was determined using analytical balance, and average weight for each formulation was calculated. The spread ratio, an important quality parameter characterized as the width of crackers per thickness was measured following AACC International Method 10-50.05 [36]. This parameter is the primary scale for baking performance and end-product quality [39].

$$\text{Spread Ratio} = \frac{\text{Width}}{\text{Thickness}} \quad \ldots\ldots\ldots\ldots\ldots\ldots\ldots\ldots\ldots\ldots(10)$$

Texture profile of biscuit (in term of hardness represented by the peak force) was determined using Texture Profile Analysis (TPA). A calibrated texture analyzer (TA. XTplus, Stable micro system Ltd, UK) fitted with a three-point bending rig (HDP/3PB) and 50 kg load cell was employed [40]. The method applied for the experimental study is based on the concept of Baltsaviaset al. (1997). Each biscuit was supported on 2 parallel bars separated by 40 mm and broken with a downwardly moving blade. The probe was moved at a pre test and post speeds of 1.0 mm/s, a test speed of 3.0 mm/s and a trigger force of 5 g. The highest force (N) recorded during penetration of the probe represented the hardness value. At least ten replicates of each batch were analyzed. The surface colour on the top and bottom surfaces of the biscuits was measured by using a HunterLab colorimeter (ColorFlex EZ, Hunter Associates Laboratory, Inc., USA), calibrated against white and black standard tiles.

The CIE L, *a*, b* colour space values were recorded where L* represents lightness (0 = black, 100 = white), a* represents the green-red component (negative values indicate green, positive values indicate red), and b* represents the blue-yellow component (negative values indicate blue, positive values indicate yellow). The total colour difference (ΔE) between the control and NFK-substituted biscuits was calculated using the equation 11 [41, 42]:

$$\Delta E = \sqrt{[(L - L_0)^2 + (a - a_0)^2 + (b^* - b_0)^2]} \quad \ldots\ldots\ldots\ldots\ldots\ldots\ldots\ldots(11)$$

where L_0, a_0, and b_0 are the colour values of the control biscuit. Furthermore, the chemical composition of the final biscuit products, including moisture, ash, crude protein, crude fat, crude fiber, and total carbohydrate content, was determined using the same official AOAC [23].

pH were determined using pH meters (Model: HannaHI-255, USA). The concentrations of anions such as Cl^- , and SO_4^{2-} , and were analyzed by ion chromatography (Model: HIC-10A super, Shimadzu, Japan), while PO_4^{3-} was investigated using an UV–Vis spectrophotometer (model: UV-1650PC, Shimad Zu, Japan).

2.6. Sensory Evaluation

The sensory acceptability of biscuit formulations with *Nypa fruticans* kernel (NFK) substitution was evaluated through an affective consumer acceptance test. A panel (n=120) of untrained consumers, recruited based on their frequency of consuming baked goods and absence of relevant allergies, participated [43, 44]. Sensory testing was conducted [10/01/2024 to 10/01/2025] in a dedicated laboratory with isolated booths, following International Standards (ISO, 2007) [45]. The environment was maintained under controlled white light and a constant ambient temperature of 22 ± 1°C to minimize external influence. A complete block design was used, where all panelists evaluated all samples—including the 0% NFK control and the experimental treatments in a monadic and randomized order. Quarter-biscuit pieces were laid out on white, odourless plastic plates at room temperature and were coded with a three-digit code in order to avoid bias. Subjects rinsed their palate with distilled water between evaluations. The consumers scored the appearance, color, aroma, flavor, texture and aftertaste on a nine-point hedonic scale (1 = dislike extremely; 9 = like extremely) [46, 47]. This approach effectively confirmed the strong positive effect of NFK incorporation on consumer liking and perception.

2.7 **Microbiological Analysis and Shelf-Life Study**

The microbiological safety and stability of the developed low-fat biscuits were followed during storage to establish shelf-life. Study of shelf-life Shelf-life study was performed by keeping the packed biscuit samples processed from each formulation (control and NFK-substituted) at room temperature conditions (25 ± 2°C, 65% relative humidity) for a period of six months. The microbiological analyses of the bread samples were conducted immediately after baking and cooling (time zero) and repeated at monthly intervals to monitor microorganism proliferation in accordance with the standard methods recommended by APHA, 2018 [28] and ICMSF, 2018 [48]. The examination included the determination of total viable microbial load, indices for hygiene and fecal contamination, as well as directed search for bacterial pathogens. For all the analyses, a 10 g sample of each homogenized biscuit was aseptically weighed and placed in a sterile stomacher bag

containing 90 mL of buffered peptone water (BPW; Himedia, India) to have a 1:10 stock dilution. The homogenate was mixed in the stomacher (Model 400, Seward Ltd., UK) for 2 min to achieve a homogeneous suspension. Decimal dilutions (10^{-1} to 10^{-5}) of the first homogenate were prepared in sterile 0.1% peptone water for plating. The Total Viable Count (TVC) of heterotrophic bacteria, a primary parameter reflecting the overall microbiological quality and spoilage of fish, was enumerated using spread plating technique on Plate Count Agar (PCA). Briefly, 0.1 mL of proper dilution were spread on the surface of pre-poured PCA plates (three replicates) and incubated aerobically at 36°C for 48 ± 2 h [49]. After incubation, colonies were counted and the amounts were calculated as colony forming units per gram of sample (CFU/g) according to formula No. 12 [50]:

$$\text{TVC (CFU/g)} = \frac{\text{Number of colonies} \times \text{Dilution Factor}}{\text{Volume of culture plated (mL)}} \ldots\ldots\ldots\ldots\ldots\ldots\ldots\ldots\ldots\ldots\ldots\ldots (12)$$

Quantitative microbial risk assessment was carried out by using a three-tube Most Probable Number (MPN) method for coliforms, indicator organisms and others. The presumptive test was performed, inoculating 1 mL of the 10^{-1}, 10^{-2}, and 10^{-3} dilutions into a test tube with Lauryl Tryptose Broth (LTB) with Durham's tubes. The tubes were kept in a 37°C incubator for total coliforms and the incubation temperature for fecal coliform was 44.5°C. Tubes that demonstrated gas production and turbidity were regarded as positive. These strains were subsequently subcultured into Brilliant Green Bile Broth (BGBB) for a confirmed test, and incubated as above. The MPN value per 100 g was calculated using standard MPN tables for the number of positive tubes at each dilution.

Furthermore, tested for major foodborne pathogens in the samples statements of selective. For Escherichia coli, 0.1 mL of homogenate was plated on Eosin Methylene Blue (EMB) agar and incubated at 37°C for 24h, bacteria consisting in metallic green sheen colonies were considered as presumptive. For Salmonella and Shigella spp., selective enrichment was carried out and plated on *SS agar,* before being incubated at 37°C for 24 h. The characteristic colorless colonies (for *Salmonella*) and Black centered ones for *Shigella* were observed. *Vibrio spp.* the detection was carried on Thiosulfate-Citrate-Bile Salts-Sucrose (TCBS) agar, at 37 °C for 24 h, and yellow-green colonies indicated the fermentation of sucrose. The presence of these pathogens was a critical component for product safety in 25 g of sample.

2.8. Statistical Analysis

All chemical and sensory values of biscuits prepared were statistically analyzed to compare the difference among biscuit formulations by a Completely Randomized Design (CRD) [52, 53]. Results of all laboratory analyses (n=3) are expressed as mean ± standard deviation. Statistical significance was calculated with one-way analysis of variance (ANOVA) using OriginPro software [54] with $p < 0.05$. Mean separations were done by Duncan's Multiple Range Test (DMRT) for the significant factors [55]. Second, sensory data (n=120 panelists) were analyzed with ANOVA, along with Tukey's HSD for multiple comparisons which is efficient in case of large and equal sample size [56].

The equation of the statistical model for the experiment becomes:

$$Yij = \mu + \tau i + \varepsilon ij \quad (13)$$

where Yij is the growth status of each child at baseline, indicating whether children presented with stunting or not.

where: Yij is j-th observation of the i-th treatment, μ is overall mean, τi is treatment effecto fthe i-treatment and εij is the random error associated to j-th observation i- thtreatment assumed to be independentl and no rmally distributed with median given for zero and homogeneous variance.

3 Results and Discussion

3.1 Physical Properties of Biscuits

The incorporation of NFK flour significantly altered key sensory attributes, which are important factors determining acceptance by consumers and quality of products (Table 1). Most pronounced was the alteration of dimension-related features. Biscuit width increased significantly with increasing level of substitution of NFK from 52.10 mm in the control to 55.15mm in biscuit incorporated with 30% NFK. On the other hand, thickness decreased significantly but slowly from 8.20 mm to 7.55 mm.

Table 1 Physical characteristics of biscuits made from NFK flour.

Parameter	Control (0% NFK)	10% NFK	20% NFK	30% NFK
Width (W, mm)	52.10 ± 0.45^{c}	53.25 ± 0.38^{b}	54.80 ± 0.52^{a}	55.15 ± 0.49^{a}
Thickness (T, mm)	8.20 ± 0.15^{a}	7.95 ± 0.12^{ab}	7.70 ± 0.18^{b}	7.55 ± 0.14^{c}
Spread Ratio (W/T)	6.35 ± 0.12^{c}	6.70 ± 0.09^{b}	7.12 ± 0.15^{a}	7.30 ± 0.11^{a}
Weight (g)	12.50 ± 0.25	12.45 ± 0.30	12.55 ± 0.22	12.48 ± 0.27
Hardness (N)	35.20 ± 1.50^{a}	32.80 ± 1.25^{b}	29.50 ± 1.40^{c}	27.90 ± 1.35^{d}
Color (L*)	65.30 ± 1.20^{a}	62.15 ± 1.05^{b}	58.90 ± 1.30^{c}	55.40 ± 1.15^{d}
Color (a*)	4.50 ± 0.25^{c}	5.20 ± 0.30^{b}	5.85 ± 0.22^{a}	6.10 ± 0.28^{a}
Color (b*)	25.80 ± 0.80^{a}	24.20 ± 0.75^{b}	23.10 ± 0.85^{c}	22.50 ± 0.70^{c}

All values are presented as mean ± SD (n=3 for dimensions, weight, hardness; n=10 for color). Means in the same row with different superscript letters are significantly different ($p < 0.05$) according to Duncan's Multiple Range Test.

The spread ratio increased from 6.35 in the control to 7.30 in the biscuit formulation containing 30% NFK flour. This increased spread is a desirable characteristic for biscuits, contributing to a more aesthetically pleasing appearance. The effect is likely due to the dilution of wheat gluten and the unique water absorption capacity and rheological properties of NFK fibre. These factors may reduce dough elasticity and increase extensibility during the early stages of baking, prior to crust formation, resulting in greater lateral flow. This result is consistent with those recently reported on date seed powder fortified biscuits [57] and may be due to the breakdown of gluten network and changes in dough rheology.

The textural attribute, hardness was highly affected by NFK addition; it decreased significantly from 35.20 N in the control to 27.90 N in the biscuit containing 30% NFK. This decrease in fracture force implies a softer and more brittle texture, which is commonly accepted by consumers. The softening may have been due to gluten dilution, as the formation of NFK particles can disrupt the protein network, interference of NFK particles in the starch-protein matrix and dietary fiber lubricity. This result is in agreement with a study on jackfruit seed flour [58], which also showed lower hardness values which is indicative of the disruption of gluten-starch network due to increased dietary fiber and hence less compact structure. This softening interaction is desirable because it describes a typical technical problem with low-fat bakery products, i.e., keeping the tenderness and preventing a tough or firm texture.

The surface characteristics of the biscuits, as measured by CIE Lab, were appreciably influenced by NFK incorporation. The LL values (65.30 to 55.40) were significantly ($p < 0.05$) lower, a* (color redness; up to 4.50–6.10) was higher and b* (color yellowness; up to 25.80–22.50) was lower in red pepper paste for each treatment than that of the control group with increasing irradiation doses from GIPT and LIAT filed radiations which represented darker color stained product compared with the control groups in all cases empowered by irradiation treatments as well as doses applied in these studies ranging from SIPT =300 Gy – LIAT =750 Gy. This change to a dark brown reddish hue is due to the natural color and chemistry of the endosperm of NFK flour.

The higher amounts of fiber and sugar present in the MHS may have favored Maillard reaction and caramelization during baking at 180°C, which could favour the formation of melanoidins as well as other coloured products. Such darkening effect has also been reported for fortified biscuits containing mango seed kernel powder [59]. It is also worth mentioning that this darker color was sensory favorable with high scores, so it must had been perceived as appealing and associated like a whole grain product full of nutrients. In contrast, biscuit weight was consistent among formulations, which suggest that NFK substitution did not affect the baking loss or dough handling. In summary, the inclusion of NFK flour improved the biscuits' physico-sensory properties favorabvly and produced a biscuit with higher spread ratio, softer texture and optimum dark brown colour acceptable to consumers which will cater for more healthful high quality bakery products presently demanded by consumers.

3.2 Proximate and Mineral Composition of NFK Flour

The estimation (Table 2) of pH, salinity and mineral composition validates the chemical stability and fortification achieved by addition of NFK flour in biscuit formulations. Valuable minerals like calcium, iron were preserved fostering fortification and food safety. The near-neutral pH of NFK flour (6.4) was carried over into the final biscuit product (6.46), suggesting that although Maillard reaction and caramelization reactions occurred during baking, no significant acidic compounds were generated. This pH stability is also preferable for storage as it prevents the growth of numerous spoilage microorganisms-a similar pH range 6.3 -6.5 was found on rice biscuits enriched with tamarind seed powder [60].

The observed decrease in salinity may be related to the recipe itself. The large amounts of low-salt ingredients (refined wheat flour and sugar) mixed with NFK flour caused a reduction in ionic species per unit mass, as reported for mixtures similar composite food systems [61].

Table 2. Chemical Composition of Flour and Biscuits.

Parameters	Flour	Biscuits
pH	6.4	6.46
Na (mg/Kg)	125.09	241.43
K (mg/Kg)	3.75	3.5
Ca (mg/Kg)	295.2	428.4
Mg (mg/Kg)	360.3	384.1
Fe (mg/Kg)	21.5	53.3
Mn (mg/Kg)	10.3	16.7
Cd (mg/Kg)	<0.01	<0.01
Cr (mg/Kg)	1.0	1.41

Hg (mg/Kg)	<0.01	<0.01
Cu (mg/Kg)	2.7	2.94
Zn (mg/Kg)	10.1	12.9
As (mg/Kg)	<0.01	<0.01
Pb (mg/Kg)	0.76	0.85
SO_4^{2-} (mg/Kg)	9.8	45.25
PO_4^{3-} (mg/Kg)	70.6	63.2
Cl^- (mg/Kg)	8.17	3.66

The addition of NFK significantly enhanced the mineral content of biscuits. Importantly, concentrations of toxic minerals such Cd, Hg and As were not determined (LOQ: 0.01 mg/kg) in either flour or biscuits; however, the concentration Pb slightly increased from 0.76 to 0.85 mg/kg a.u. These values are far lower than the maximum allowed limits of Codex Alimentarius Commission (e.g. 0.1 mg/kg for Pb in cereals) and indicate the safety compliance of NFK to be used as food [26].

An abundant enrichment of key elements was obtained from this analysis. The content of Na went up from 125.09 to 241.43 mg/kg, being due to the leavening agent (sodium bicarbonate) and salt (NaCl) used in the formulation of rico bread. On the other hand, K levels slightly diminished from 3.75 to 3.50 mg/kg and may have resulted from its thermal degradation or leaching; otherwise K losses are likely also nutritionally irrelevant as a result of its low content in samples. The profile of bone-building minerals [62] in particular was improved with significant increases seen: Ca from 295.2 to 428.4 mg/kg and Mg from 360.3 to 384.1 mg/kg for example). Among these, Fe registering more than two-fold increase from 21.5 to 53.3 mg/kg followed by Mn was increased from 10.3 to 16.7 mg/kg were the highest increase. This high iron absorption is very beneficial, especially considering that many people around the world suffer from a lack of this element. The iron content of the NFK biscuit is significantly higher than that reported in biscuits fortified with quinoa (28–35 mg/kg) or amaranth (32–40 mg/kg) flour [63]. Zn and Cu levels also increased slightly, to 12.9 mg/kg and 2.94 mg/kg respectively.

The anions analysis showed that some changes were significantly related to the chemistry of recipe. The SO_4^{2-} also showed a remarkable increment from 9.8 mg/kg to 45.25 mg/kg. This increase could be due to heat-induced decomposition of sulfur-containing amino acids such as those present in eggs in the presence of the leavening agent. On the other hand, PO_4^{3-} decreased from 70.6 mg/kg to 63.2 mg/kg might be attributed that it is involved in chemical reaction or bonded with other minerals. A significant reduction in Cl^- content from 8.17 mg/kg to 3.66 mg/kg

was achieved. This is surprising in the presence of NaCl, and this decrease might be related to volatilization of chloride compounds or other interactions taking place during baking.

Biscuits made from NFK flour are successful formulations for the concentration of nutritionally important minerals specifically Fe, Ca, Mg and Zn in the end product. These results indicate that these biscuits are safe for consumption and can be used as dietary source for the essential elements [59]. The amount of mineral enrichment obtained with NFK is even higher than for the other novel fortifiers discussed in recent literature, further indicating its high potential as a functional ingredient to address micronutrient malnutrition [58].

3.3 **Proximate Composition of Developed Biscuits**

Application of the raw nfk flour to produce baked biscuits included major physicochemical changes (Table 3), which is in line with increasing interest for sustainable functional food ingredients worldwide. The low moisture content in baked biscuit (2.91%) as compared to raw flour (7.1%) was due to loss of water during baking that lead to concentration of other components and gave good degree of microbial stability during storage). This figure is similar to that found in biscuits from other flours such as mango kernel (2.8–3.5%) and watermelon rind powder (3.1–3.8%) [36, 59] and also within the desirable range for products with long shelf-life.

It should be noted that when the ash content is reduced from 2.3% to 1.1%, this does not represent a loss of minerals, but rather a dilution due to the contribution of low-ash ingredients such as refined wheat flour and sugar which is common in composite baking [63, 64]. The amount of protein was similar over the seasons and just decreased slightly from 3.0% to 2.8%. This high stability suggests low protein denaturation or Maillard reaction, possibly because of the high carbonhydrate/protein ratio in the formulation. This range was similar to those other fruit kernel flours such as the date seed powder (2.5–3.2%) [57].

A primary nutritional enhancement was a >38% decrease in fat, from 6.3 to 3.9%, which is consistent with the industry trend towards lower-fat indulgent products. Carbohydrate content increased from 78.11% to 84.95%, as a consequence of the 25% sugar addition that is normally introduced in short dough biscuit production [65]. This is well-founded on the bases for significant increase of all sugar fractions; total sugars (11.1 20.1 mg/100g), reducing sugars (9.2 to 15.3 mg/100g) and non-reducing sugars (1.9 to 4.8 mg/100g). The increase in reducing sugars becomes

more important since these are crucial reactants of the Maillard reaction that produced the brown color and baked flavor which was well accepted by the sensory panel.

Table 3: Chemical Composition of Nipa flour and Biscuit

Component	Flour	Biscuit
Moisture (%)	7.1	2.91
Ash (%)	2.3	1.1
Protein (%)	3.0	2.8
Fat (%)	6.3	3.9
Crude fibre (%)	0.19	1.35
Carbohydrate (%)	78.11	84.95
Total Sugar (mg/100g)	11.1	20.1
Reducing Sugar (mg/100g)	9.2	15.3
Non Reducing Sugar (mg/100g)	1.9	4.8
Total Energy (mg/100g)	390.1	505.14
Vit-C (mg/100g)	2.5	1.2
Vit-A (mg/100g)	20.5	17.63
$NaHCO_3$ (%)	0.3	3.0
Salt (NaCl) (%)	0.01	0.05
Egg (%)	0.0	4.0%

An outstanding nutritional enhancement was the over seven-fold increment of crude fibre, from 0.19% in flour to a final biscuit figure of 1.35%. This focusing effect, together with the loss of moisture during baking, leads to a fibre level which is just clear of most commercial refined flour biscuits that are lower than 1% fibre. In addition, surpassing a fibre content of 1.2% is referred to as an important cut-off point for functional foods also within research on pseudocereal-enriched biscuits [62], which supports the value of dietary suggestion about daily intake of fibre. The total energy content rose from 390.1 kcal/100g to 505.14 kcal/100g using modified Atwater factors. This energy density is similar to that of others fortified biscuits, for example made from jackfruit seed flour (495-510 kcal/100g) [58] and represents the caloric contribution of the added sugars.

Unsurprisingly, the heat-labile vitamins were destroyed. Vitamin C was reduced by 52% (from 2.5 mg/100g to 1.2 mg/100g), which agreed with the heat- and oxidation-sensitivity of vitamin C during baking as reported previously [23]. Vitamin A also decreased with 14% (from 20.5 to 17.63 mg/100g) due to isomerization and oxidative losses, respectively. The end concentrations of these key recipe ingredients (sodium bicarbonate 3.0%, salt 0.05% and egg 4.0%) confirm the correct application of the experimental formula with respect to typical levels for leavening, taste, and structure in biscuit preparation.

It was concluded that the NFK biscuit optimally retained the nutritional attributes of the kernel with a high fibre content and low fat level, relevant to recent health trends. The physicochemical changes recorded by proximate analysis are reported for last literature on biscuits enriched with new and sustainable ingredients, validating the incorporation of NFK as functional food ingredient.

3.4 **Sensory Evaluation Results**

The sensory results of biscuits with various levels of NFK powder (Table 4) are very important to know about acceptability by consumer and market value the product. Formulations (A–E): All formulations (A-E) received relatively high hedonic ratings (7.85 to 8.95, using a 9-point hedonic scale) for taste, texture, color, odor and overall acceptability (like very much to like extremely). It is interesting to note that the profile for NFK 100 (as reflected by E in this case) received the highest value influenced other sensory characterization, thus demonstrating that NFK was not only a flavorful ingredient but free of off-flavors frequently seen with many plant based fortificants. This is in comparison to legume-flour-enriched biscuits that often have beany or bitter tastes which reduce acceptability and require flavour-masking agents [65, 66].

Textural constants similarly achieved high scores (≥8.0), comparable to that by mango kernel powder biscuits [67] and higher than in many high-fibre biscuits with hard or gritty texture sensations. The smallinease in crispness due to processing from underact A (8.0) of however, illustrates the water binding capacity oi01 fibre. Nevertheless, this slight decrease did not compromise overall acceptability (7.95 for E), suggesting that NFK can be added without the severe textural.

Table 4: Organoleptic test result of Biscuits.

Parameters	A	B	C	D	E
Taste	8.1	8.05	8.0	8.0	8.95
Texture	7.9	8.0	7.95	7.9	7.85
Color	8.2	8.1	8.15	8.05	8.05
Odor	8.05	8.05	8.1	8.05	8.0
Mouth Feel	8.15	8.1	8.1	8.05	8.1
Crispiness	8.0	7.95	7.9	7.9	7.85
Acceptability	8.0	7.95	7.95	7.9	7.95

Rating for color (8.05–8.2) and odour (8.0–8.1) was high and it indicates that desired browning without rancidity took place along with pleasant aromas. Most notably, total acceptability scores (7.9-8.0) were similar between substitution levels. This supports that NFK has indeed significantly

and effectively added nutritional value without negatively affecting sensory quality, being an important advantage that meets clean-label trends and performs better than many other novel ingredients that experience acceptability reduction beyond a 10–15% substitution rate [68]. In summary, the data presented clearly confirms NFK to be a nutritionally rich material possessing good organoleptic properties, offering potential for manufacture of consumer appealing biscuits that are acceptable in terms of both nutritional quality and taste requirements in today's market.

3.5 Shelf life test

Microbiological examination of NFK-exported biscuits, it was safe with a fairly good keeping quality during twelve months of storage (Table 5), showing the safety and stability that are ready for commercial shelf-life. Indicators of public health significance, Total Coliforms and Fecal Coliforms, consistently were below the method detection limit (1.8 MPN/100g) for all samples during this period. This lack confirms the hygienic production conditions and proves that there is sufficient killing during baking. Additionally, pathogen-specific qPCR demonstrated the lack of *E. coli, Salmonella, Shigella and Vibrio spp.* in all samples. These findings comply with and even surpass the strict microbiologic standards set for ready-to-eat foods by Codex Alimentarius Commission [48], which usually recommend absence of these pathogens in a 25g sample for being confirmed that product is safe.

Table 5: Microbial status (shelf life) of *NFK Biscuits*.

ID	HBC log (CFU/gm) ±SD	TC (MPN/100gm)	TFC (MPN/100gm)	*E. coli*	*Salmonella*	*Shigella*	*Vibrio*
A	5.505±0.326	<1.8	<1.8	Absent	Absent	Absent	Absent
B	3.602±0.421	<1.8	<1.8	Absent	Absent	Absent	Absent
C	3.301±0.214	<1.8	<1.8	Absent	Absent	Absent	Absent
D	2.698±0.09	<1.8	<1.8	Absent	Absent	Absent	Absent
E	2.301±0.121	<1.8	<1.8	Absent	Absent	Absent	Absent

This general decrease trend is typical of shelf-stable, low-water activity foods and can be viewed as a positive feature. It varies immensely from high- moisture foods that usually show rising levels of microorganisms as they develop towards spoilage. The low and decreasing microbial counts, the lack of pathogenic and indicating organisms verify the intrinsic microbiological stability of the low-fat biscuit enriched with NFK. The ingredients of the product, together with the processing steps and low water activity form a microbiologically controlling food matrix which guarantees safety during an estimated shelf life of 1 year. Indeed, such results are in accordance with previous

research on the shelf-life of biscuits fortified using other plant materials that also use low water activity as one of the leading preservation factors [62]. The microbiological studies reveal that the addition of NFK flour gave rise to a well-fortified, low-fat biscuit having excellent microbial safety and capable to retain its organoleptic properties for at least 1 year when stored under normal conditions. NFK is found to be a potential and safe component for the functional food industry, meeting well both the nutritional and food security reasons.

4. Conclusion

In the current study, we have proofed that NFK flour is a potential and sustainable functional alternative to produce low-fat and high in nutritional value biscuits. The total analysis indicates that NFK-substitution by wheat flour leads to a considerable improvement of the quality of biscuits fortified and enriched with essential mineral elements (Fe, Ca and Mg) such as which offsets the common not sufficient consumption level about microelements. The incorporation of NFK had a positive impact on key physical properties leading to cakes with higher spread ratio, better texture desirability, and so after crumb softness and color which were preferred by the consumer in sensory panel analysis. Crucially, the sensory outcomes demonstrated that NFK incorporation up to 30% resulted in biscuits accepted on the basis of taste and texture, as well as overall acceptance (i.e., not having any off-flavors associated with plant-based fortificants). Furthermore, the good microbiological stability after storage for 12 months (absence of any pathogen and low counts in microbes) reiterated the heat-stability and sound shelf-life safety offerings of the product from a commercial perspective. By valorizing an underused natural resource, it also helps to promote innovative product development for the gluten-free and clean-label bakery industry and this ferment provides opportunities for reduced waste, sustainable resource usage and enhancing food security which offer economic profitability as well as social benefits in coastal areas where nipa palm grows.

Commercially, implementation of integrated nutritional labeling and effective marketing strategies that would underscore the low fat content, high mineral contents as well as sustainable source will be important for consumer awareness and market acceptance. Lastly, policy-based interventions need to be promoted for promoting sustainable *Nypa fruticans* cultivation and collection using an ethical sourcing model that would help in providing economic benefits to the local populations as well as contribute in mangrove conservation activities. This integrated approach from research

through to market place will guarantee that this new functional ingredient has the opportunity to fulfil its potential in assisting global food and nutrition security.

Acknowledgments

The authors are grateful to the authority of the Institute of National Analytical Research and Service (INARS), BCSIR, Dhaka, 470 Bangladesh for support for conducting this research work. Thanks to Ministry of Science and Technology, The People's Republic of Bangladesh for providing analytical, technical, and other logistic support for conducting this research work.

Funding: King Khalid University's Deanship of Scientific Research for funding via grant RGP 2/157/46.

Data availability statement: All data are available in the manuscript.

Authors credit statement

Md. Ripaj Uddin: Conceptualization, Methodology, Formal analysis, Investigation, Data curation, Project Administration, Validation, Writing-original draft preparation, Software, Writing-Reviewing and editing; Md. Khairul Islam and Abubakr M. Idris: Funding Acquisition, Resources, Writing-Reviewing and editing; Muhammad Abdullah Al-Mansur, Sirajum Monira, Md. Salim Khan: Resources, Writing-Reviewing and editing; Md. Selim Reza, Md Hamedul Islam, Mehedi Hasan, Sharmin Ahmed: Methodology, Formal analysis.

5. References

[1] Arepally, D., Reddy, R. S., Goswami, T. K., & Datta, A. K. (2020). Biscuit baking: A review. In *LWT* (Vol. 131). https://doi.org/10.1016/j.lwt.2020.109726

[2] Birch, C. S., & Bonwick, G. A. (2019). Ensuring the future of functional foods. In *International Journal of Food Science and Technology* (Vol. 54, Issue 5). https://doi.org/10.1111/ijfs.14060

[3] Mollakhalili-meybodi, N., Ehrampoush, M. H., Hajimohammadi, B., & Mosaddegh, M. H. (2023). Formulation optimization of functional wheat bread with low glycemic index from technological and nutritional perspective. *Food Science and Nutrition*, *11*(1). https://doi.org/10.1002/fsn3.3060

[4] Wang, Y., & Jian, C. (2022). Sustainable plant-based ingredients as wheat flour substitutes in bread making. In *npj Science of Food* (Vol. 6, Issue 1). https://doi.org/10.1038/s41538-022-00163-1

[5] Baker, W. J., & Dransfield, J. (2016). Beyond Genera Palmarum: progress and prospects in palm systematics. In *Botanical Journal of the Linnean Society* (Vol. 182, Issue 2). https://doi.org/10.1111/boj.12401

[6] Christenhusz, M. J. M., & Byng, J. W. (2016). The number of known plants species in the world and its annual increase. In *Phytotaxa* (Vol. 261, Issue 3). https://doi.org/10.11646/phytotaxa.261.3.1

[7] Islam, M. N., Dana, N. H., Rahman, K. S., Hossain, M. T., Ahmed, M. U., & Sadig, A. (2020). Nypa fruticans Wurmb leaf collection as a livelihoods strategy: a case study in the Sundarbans Impact Zone of Bangladesh. *Environment, Development and Sustainability*, *22*(6). https://doi.org/10.1007/s10668-019-00438-w

[8] Islam, S. M. D.-U., & Bhuiyan, M. A. H. (2018). Sundarbans mangrove forest of Bangladesh: causes of degradation and sustainable management options. *Environmental Sustainability*, *1*(2). https://doi.org/10.1007/s42398-018-0018-y

[9] Hamilton, L. S., & Murphy, D. H. (1988). Use and management of Nipa palm (Nypa fruticans, arecaceae): a review. *Economic Botany*, *42*(2). https://doi.org/10.1007/BF02858921

[10] Bobrov, A. V. F. C., Lorence, D. H., Romanov, M. S., & Romanov, E. S. (2012). Fruit development and pericarp structure in Nypa fruticans Wurmb (Arecaceae): A comparison with other palms. *International Journal of Plant Sciences*, *173*(7). https://doi.org/10.1086/666668

[11] Akpabio, U. D., Essien, U. C., & Eka, O. U. (2007). Chemical composition of the kernal of Nypa fruticans Nypa Palm) and its application in confectionary preparation. *Global Journal of Pure and Applied Sciences*, *13*(4), 513–516.

[12] Ulyarti, Nazarudin, & Sari, D. W. (2017). The study of functional properties of Nypa fruticans flour. *AIP Conference Proceedings*, *1823*. https://doi.org/10.1063/1.4978100

[13] Mishra, A., Devi, M., & Jha, P. (2015). Development of gluten free biscuits utilizing fruits and starchy vegetable powders. *Journal of Food Science and Technology*, *52*(7). https://doi.org/10.1007/s13197-014-1521-5

[14] Di Cairano, M., Galgano, F., Tolve, R., Caruso, M. C., & Condelli, N. (2018). Focus on gluten free biscuits: Ingredients and issues. In *Trends in Food Science and Technology* (Vol. 81). https://doi.org/10.1016/j.tifs.2018.09.006

[15] [1] M. M. Haque, M. S. Rana, M. S. H. Khan, M. N. U. Chowdhury, and A. B. M. S. Hossain, "Proximate composition and mineral profiling of *Nypa fruticans* Wurmb. (fruit and endosperm) collected from the southern district of Bangladesh," *Journal of Agriculture and Food Research*, vol. 7, p. 100271, 2022.

[16] J. F. Kennedy, J. S. Sandhu, and D. T. Southgate, "Structural data for the carbohydrate of ivory nut (*Phytelephas macrocarpa*)," *Carbohydrate Research*, vol. 90, no. 1, pp. 139-144, 1981.

[17] A. A. Mariod, M. S. Abdelwahab, S. I. G. Abdelmageed, and F. H. Jahurul, "Characteristics, composition and thermal properties of oils from two species of date palm stone (*Phoenix*

dactylifera L.) from Sudan," *Journal of the American Oil Chemists' Society*, vol. 89, no. 11, pp. 2107-2113, 2012.

[18] S. M. Abdul-lah, A. N. A. Alawy, and H. H. Al-Masoodi, "Utilization of mango seed kernel as a source of oil and food flour: A review," *IOP Conference Series: Earth and Environmental Science*, vol. 761, no. 1, p. 012085, 2021.

[19] O. L. Erukainure, O. V. Oke, A. J. Ajiboye, and R. N. Okafor, "Nutritional qualities and phytochemical constituents of *Clausena anisata* hook, a spice plant," *Nuts and Seeds in Health and Disease Prevention*, pp. 417-424, 2011.

[20] T. A. Shittu, R. A. Lawal, and M. O. A. Adewusi, "Functional properties of pretreated okra (*Abelmoschus esculentus*) seed flour," *Food Science and Technology International*, vol. 18, no. 4, pp. 389-398, 2012.

[21] M. K. Kaushik, S. Kumar, and R. Kumar, "Effect of particle size on functional and physicochemical properties of defatted flaxseed flour," *Journal of Food Science and Technology*, vol. 57, no. 8, pp. 3059-3066, 2020.

[22] M. A. Hossain, M. S. R. Siddique, and S. H. Rahman, "Chemical compositions of oil and cake of *Nypa fruticans* Wurmb," *Bangladesh Journal of Scientific and Industrial Research*, vol. 44, no. 4, pp. 437-442, 2009.

[23] AOAC International. (2016). *Official Methods of Analysis of AOAC International* (20th ed.). Gaithersburg, MD, USA.

[24] Nielsen, S. S. (2017). *Food Analysis Laboratory Manual* (3rd ed.). Springer International Publishing.

[25] Merrill, A. L., & Watt, B. K. (1973). *Energy Value of Foods: Basis and Derivation*. USDA Agriculture Handbook No. 74. United States Department of Agriculture.

[26] Codex Alimentarius. (1995). *Codex Standard for Wheat and Durum Wheat* (Codex Stan 199-1995). FAO/WHO Food Standards Programme.

[27] Food and Agriculture Organization (FAO). (2003). *Food Energy - Methods of Analysis and Conversion Factors*. FAO Food and Nutrition Paper 77. Rome: FAO.

[28] American Public Health Association (APHA), American Water Works Association (AWWA), Water Environment Federation (WEF). (2018). *Standard Methods for the Examination of Water and Wastewater* (23rd ed.). Washington, D.C.: APHA Press.

[29] ISO/IEC 17025:2017. (2017). *General requirements for the competence of testing and calibration laboratories*. International Organization for Standardization (ISO), Geneva, Switzerland.

[30] PerkinElmer, Inc. (2017). *Analytical Methods for Atomic Absorption Spectroscopy*. Waltham, MA, USA.

[31] Jamal, A. H. M. S. I. M., Ahmed, S., Akter, S., Nahar, A., Sultana, R., Uddin, M. R., ... & Hasan, M. (2020). Spatial variation of rainwater quality parameters at Khulna City of Bangladesh. *Journal of Environment Pollution and Human Health*, *8*(2), 49-54.

[32] Azad, A. R., Karim, M. R., Rakib, M. R. J., & Uddin, M. R. (2025). Assessment of heavy metal pollution and spatial distribution in waterfalls of Chattogram District, Bangladesh: Implications for drinking and irrigation purposes. *Environmental Advances*, *19*, 100613.

[33] Uddin, M. R., Siddique, M. A. B., Sultana, S., Bithi, U. H., Akter, N., Idris, A. M., ... & Khandaker, M. U. (2024). Techno-economic assessment and innovative production of nutrient-rich jam, jelly, and pickle from Sonneratia apetala fruit. *PloS one*, *19*(12), e0311846.

[34] Manley, D. (2011). *Manley's technology of biscuits, crackers and cookies* (4th ed.). Woodhead Publishing.

[35] Pareyt, B., & Delcour, J. A. (2008). The role of wheat flour constituents, sugar, and fat in low moisture cereal based products: A review on sugar-snap cookies. *Critical Reviews in Food Science and Nutrition*, 48(9), 824-839.

[36] AACC International. (2010). *Approved Methods of Analysis* (11th Ed.). Method 10-50.05: Baking Quality of Cookie Flour. St. Paul, MN.

[37] Gallagher, E., O'Brien, C. M., Scannell, A. G. M., & Arendt, E. K. (2003). Evaluation of sugar replacers in short dough biscuit production. *Journal of Food Engineering*, 56(2-3), 261–263.

[38] Jacob, J., & Leelavathi, K. (2007). Effect of fat-type on cookie dough and cookie quality. *Journal of Food Engineering*, 79(1), 299–305

[39] Baltsavias, A., Jurgens, A., & van Vliet, T. (1997). Rheological properties of short-doughs at large deformation. *Journal of Cereal Science*, 26(3), 289-301.

[40] Pareyt, B., Talhaoui, F., Kerckhofs, G., Brijs, K., Goesaert, H., Wevers, M., & Delcour, J. A. (2009). The role of sugar and fat in sugar-snap cookies: Structural and textural properties. *Journal of Food Engineering*, 90(3), 400-408.

[41]Laguna, L., Salvador, A., Sanz, T., & Fiszman, S. M. (2011). Performance of a resistant starch rich ingredient in the baking and eating quality of short-dough biscuits. *LWT - Food Science and Technology*, 44(3), 737-746.

[42] Pathare, P. B., Opara, U. L., & Al-Said, F. A. J. (2013). Colour Measurement and Analysis in Fresh and Processed Foods: A Review. *Food and Bioprocess Technology*, 6(1), 36–60.

[43] Lawless, H. T., & Heymann, H. (2010). *Sensory Evaluation of Food: Principles and Practices* (2nd ed.). Springer Science & Business Media.

[44] Meilgaard, M. C., Carr, B. T., & Civille, G. V. (2007). *Sensory Evaluation Techniques* (4th ed.). CRC Press.

[45] ISO 8589:2007. (2007). *Sensory analysis — General guidance for the design of test rooms*. International Organization for Standardization, Geneva, Switzerland.

[46] Peryam, D. R., & Pilgrim, F. J. (1957). Hedonic scale method of measuring food preferences. *Food Technology*, 11(9), 9-14.

[47] Stone, H., Bleibaum, R. N., & Thomas, H. A. (2012). *Sensory Evaluation Practices* (4th ed.). Academic Press.

[48] International Commission on Microbiological Specifications for Foods (ICMSF). (2018). *Microorganisms in Foods 7: Microbiological Testing in Food Safety Management* (2nd ed.). Springer International Publishing.

[49] ISO 4833-1:2013. (2013). *Microbiology of the food chain — Horizontal method for the enumeration of microorganisms — Part 1: Colony count at 30 °C by the pour plate technique*. International Organization for Standardization.

[50] ISO 21528-2:2017. (2017). *Microbiology of the food chain — Horizontal method for the detection and enumeration of Enterobacteriaceae — Part 2: Colony-count technique*. International Organization for Standardization.

[51] Jay, J. M., Loessner, M. J., & Golden, D. A. (2005). *Modern Food Microbiology* (7th ed.). Springer Science + Business Media, Inc.

[52] Steel, R. G. D., Torrie, J. H., & Dickey, D. A. (1997). *Principles and Procedures of Statistics: A Biometrical Approach* (3rd ed.). McGraw-Hill.

[53] Montgomery, D. C. (2017). *Design and Analysis of Experiments* (9th ed.). John Wiley & Sons.

[54] Miller, R. G. (1997). *Beyond ANOVA: Basics of Applied Statistics*. Chapman & Hall/CRC.

[55] Duncan, D. B. (1955). Multiple range and multiple F tests. *Biometrics*, 11(1), 1–42.

[56] Tukey, J. W. (1949). Comparing individual means in the analysis of variance. *Biometrics*, 5(2), 99–114.

[57] Abbehalet, M. L., Aljahani, A. H., & Alsulais, M. H. (2023). Utilization of date seed powder as a functional ingredient in biscuit production: Impact on physicochemical and sensory properties. *Journal of Food Science and Technology*, 60(3), 1022-1031

[58] Goswami, M., & Gupta, S. (2022). Optimization of process parameters for development of jackfruit (Artocarpus heterophyllus) seed flour incorporated nutritious biscuits. *LWT - Food Science and Technology*, 154, 112785.

[59] Olagunju, A. I., Omoba, O. S., Enujiugha, V. N., Wiens, R. A., & Gough, K. M. (2021). Quality attributes and storage stability of bread and cookie formulated with mango seed kernel powder. *International Journal of Food Science & Technology*, 56(2), 795-803.

[60] Eltayeb, A. R. S. M., Ali, A. O., & Abou-Arab, A. A. (2021). Physicochemical, nutritional and sensory properties of bread and biscuits enriched with tamarind seed powder. *Journal of Food Measurement and Characterization*, 15(1), 609-620.

[61] Mrabet, A., Rodríguez-Arcos, R., Guillén-Bejarano, R., Chaira, N., Ferchichi, A., & Jiménez-Araujo, A. (2022). Dietary fiber from Tunisian common date cultivars (Phoenix dactylifera L.): Chemical composition, functional properties, and antioxidant capacity. *Journal of Agricultural and Food Chemistry*, 70(20), 5938-5949.

[62] Kaur, P., Sharma, P., & Varghese, T. (2024). Pseudocereals in Bakery Products: A Review on Nutritional Enrichment, Technological Challenges, and Future Prospects. *Trends in Food Science & Technology*, 144, 104333.

[63] Mrabet, A., Rodríguez-Arcos, R., Guillén-Bejarano, R., Chaira, N., Ferchichi, A., & Jiménez-Araujo, A. (2022). Dietary fiber from Tunisian common date cultivars (Phoenix dactylifera L.): Chemical composition, functional properties, and antioxidant capacity. *Journal of Agricultural and Food Chemistry*, 70(20), 5938-5949.

[64] Yasmine, S., Proma, R. Z., Uddin, M. R., Rahman, M. S., Islam, M. H., Al Mansur, M. A., ... & Siddiqi, M. M. A. (2025). Exploring the multifaceted roles of Sonneratia apetala and Nipa fruticans in coastal habitat restoration and bioactive properties discovery. *Phytomedicine Plus*, *5*(1), 100687.

[65] Pareyt, B., & Delcour, J. A. (2008). The role of wheat flour constituents, sugar, and fat in low moisture cereal based products: A review on sugar-snap cookies. *Critical Reviews in Food Science and Nutrition*, 48(9), 824-839.

[66] ADEOLA, A. A., SOLOLA, O., APATA, O., & OHIZUA, E. R. (2019). Assessment of some attributes of unripe cooking banana (Cardaba Musa ABB), pigeon pea (Cajanus cajan) and orange fleshed sweet potato (Ipomoea batatas) flour blends for use as complementary feeding. *Croatian journal of food science and technology*, *11*(1), 67-75.

[67] Tan, E., Binti Julmohammad, N., Koh, W. Y., Abdullah Sani, M. S., & Rasti, B. (2023). Application of ATR-FTIR incorporated with multivariate data analysis for discrimination and quantification of urea as an adulterant in UHT milk. *Foods*, *12*(15), 2855.

[68] Călinoiu, L. F., Cătoi, A. F., & Vodnar, D. C. (2019). Solid-state yeast fermented wheat and oat bran as a route for delivery of antioxidants. *Antioxidants*, *8*(9), 372.

Figure

Click here to access/download;Figure;Graphical Abstract.tif

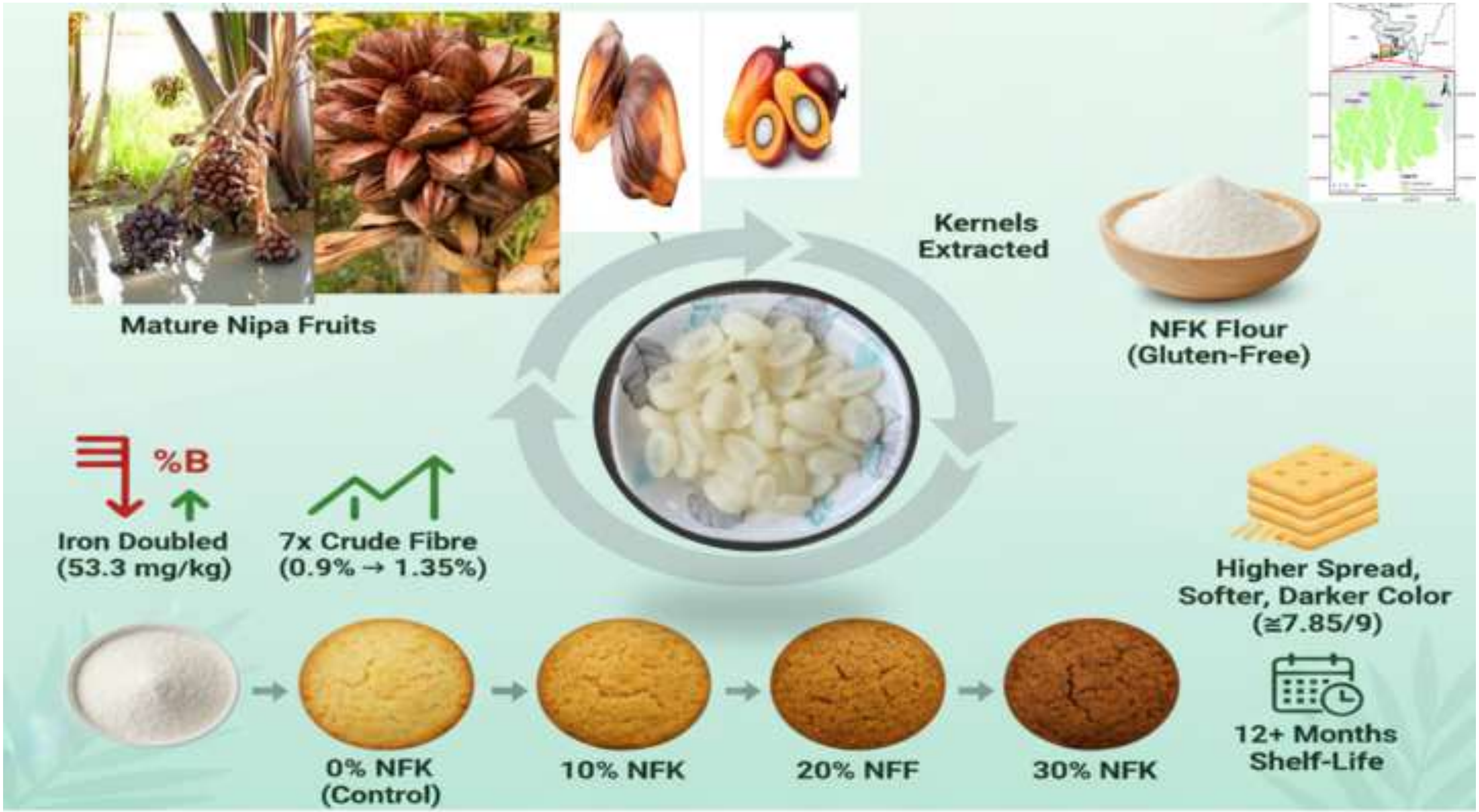

Click here to access/download;Figure;Fig. 1. Making biscuits utilizing NFK Flour..tif

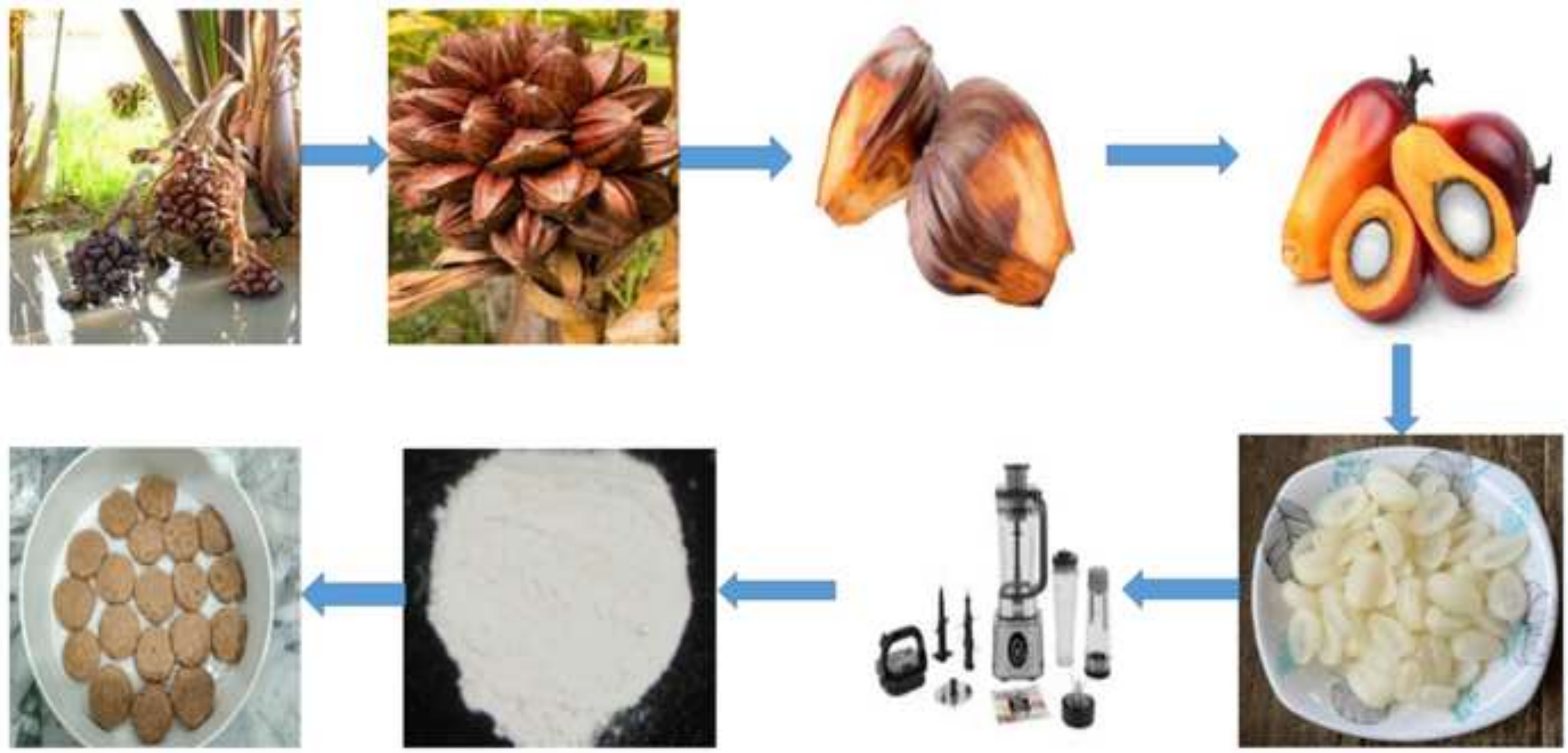

Supplement: Attachment — Submitted filename: PONE-D-25-57201_reviewer 28 November 2028.pdf [file pone.0342665.s001.pdf]
