# Supplementary material for: Nypa fruticans wurmb (Mangrove Palm) Fruit Kernel: Effect on physicochemical, sensory evaluation and shelf-life of wheat biscuits
Source: PLoS One. 2026 Feb 18;21(2):e0342665. doi: 10.1371/journal.pone.0342665 (PMC12915934; doi:10.1371/journal.pone.0342665)
Supplement: Attachment — Submitted filename: Response to Reviewer.docx [file pone.0342665.s002.docx]

The

Editor in Chief

PLOS ONE

## Subject: Submission of review comments of the manuscript: “ PONE-D-25-57201”

Dear Editor

I am submitting the reviewer comments of the manuscript “**Nypa fruticans Wurmb (Mangrove Palm) Fruit Kernel: Effect on physicochemical, sensory evaluation and shelf-life of wheat biscuits**”**.** The authors would like to start by thanking the esteemed reviewers for their thoughtful comments and suggestions. A few points have been raised for clarification and further improvement of the paper. We have addressed each comment/suggestion made by the reviewers one by one below. We have also mentioned the changes made in the revised manuscript (m/s) below. All the changes in the revised m/s have been highlighted in Yellow and track change format.

**Reviewer #1:**

**Q1**. The argument is interesting but it has to be improved. The Introduction section has to be better treated. M&M section has to be detailed. The R&D section has to be better discussed. The bibliography has to be improved. Inaccuracies in the manuscript. The manuscript has to be organised as per the instructions to authors.

***Response:*** Thank you for your suggestion. In the revised manuscript, we have **treated the introduction, detailed the methodology, and properly discussed the results** as advised.

**Q2**. Apply carefully the instructions to authors in the whole manuscript and use some recent published paper as a sample;

***Response:*** We have added some recent paper in the revised manuscript [4,5, 38-41].

**Q3.** In the title and at the first mention in the manuscript, when you write a scientific name, apply the International Botanical nomenclature and, after the species, insert the abbreviation of the Botanist (not italicised);

***Response:*** The title has been corrected in accordance with your suggestion.

Q4. Please, include the page number;

***Response:*** Thank you for your suggestion. We have added page numbers.

**Q5.** Abstract section, insert some relevant numeric result;

***Response:*** We have added the numerical data to the revised manuscript on lines 31-36.

**Q6.** Keywords section, write the keywords in alphabetical order;

***Response:*** Thank you. This has been completed.

**Q7.** Verify if to number the sections and the sub-sections;

***Response:*** We have corrected the relevant section and subsection.

**Q8**. Introduction section, lines 46-52, explain that studies were conducted to reduce the shortening by replacement of butter with a vegetable oil. Support with proper reference [X1]:

[X1] Effects of shortening replacement with extra virgin olive oil on the physical–chemical–sensory properties of Italian Cantuccini biscuits. Foods 2022, 11, 299
<https://doi.org/10.3390/foods11030299>

***Response:*** The citation has been added at line 51.

**Q9**. Introduction section, lines 48-49, this is a very important statement but poorly supported with proper references. Find, read and discuss [X2]:
[X2] Techno-functional, antioxidants, microstructural, and sensory characteristics of biscuits as affected by fat replacer using roasted and germinated chickpea (Cicer arietinum L.).
International Journal of Food Properties, 26:1, 2055-2077 (2023).
DOI: 10.1080/10942912.2023.2242602

***Response:*** The citation has been added at line 53.

**Q10**. Introduction section line 71 and in the whole manuscript, when you write the references numbers between brackets, separate them by a comma instead of a semicolon;

***Response:*** Thank you. We have now corrected it.

Q11. Introduction section line 72 and in the whole manuscript, when you write a weight, separate the numeric value and the unit: 100 g instead of 100g;

***Response:*** Thank you. We have now corrected it.

**Q12.** Sub sub-section 2.1.1., replace 02 January with 2 January;

***Response:*** Thank you. We have now corrected it.

**Q13.** Caption of figures: “Fig 1.” instead of “Fig.1.”;

***Response:*** Thank you. We have now corrected it.

**Q14**. Sub sub-section, detail the degree of ripeness of Nypa fruticans fruits. Do not write “Full ripeness” or “Industrial Fruit ripeness”. Use days after blossoming or some other recognizable degree of ripeness;

***Response:*** We have write degree of ripens in the revised version at LN 117-118.

**Q15**. Sub-section 2.2, line 122 and in the whole manuscript, tables and figures: when you write a temperature, separate the numeric value and the unit: 105 °C instead of 105°C;

***Response:*** Thank you. We have now corrected it.

**Q16.** Sub-section 2.6, and in the whole manuscript, when you write a date, apply the English form to differentiate the day of the month and the month. If you want, you can write the abbreviation of the month: Jan instead of 01;

***Response:*** It has been written in the same format.

**Q17**. Line 327 and in the whole manuscript, tables and figures: 24 h instead of 24h, separate;

***Response:*** Thank you. We have now corrected it.

**Q18**. Caption of tables, after the table number, insert a point, use some recently published paper as a sample;

***Response:*** Thank you. We have now corrected it.

**Q19**. Line 357 and in the whole manuscript, when you write a length separate the numeric value and the unit: 55.15 mm instead of 55.15mm;

***Response:*** Thank you. We have now corrected it.

**Q20**. Table 2 and in the whole manuscript, kg always in small letters;

***Response:*** Thank you. We have now corrected it.

**Q21**. Sub-section 3.3., line 449 and in the whole manuscript, the abbreviation NFK in capital letters;

***Response:*** Thank you. We have now corrected it.

**Q22.** Line 531, when you write a weight, separate the numeric value and the unit: 25 g instead of 25g, separate;

***Response:*** Thank you. We have now corrected it.

**Q23.** Table 5, the International abbreviation for grams is g and not gm;

***Response:*** Thank you. We have now corrected it.

**Q24**. Caption of table 5, after the table number, replace the colons with a point;

***Response:*** Thank you. We have now corrected it.

**Q25.** Discussion section, discuss about the interaction between the results of your different analyses;

***Response:*** In the revised manuscript (lines 630-681), we have added a discussion of the interaction between the results of the different analyses.

**Q26.** Discussion section, extend the discussion of each parameter in relation to the human diet;

***Response:*** We have added a discussion of each parameter in relation to the human diet in the revised manuscript (lines 683-699) and subsection 4.1.

**Q27.** The discussion has to be supported by your statistical data;

***Response:*** In the revised manuscript (lines 630-681), we have added a discussion of the findings, supported by statistical data.

**Q28.** Before the conclusion section insert a brief section discussing interpretation, implications and limitations of your study;

***Response:*** A discussion of the interpretation, implications, and limitations of your study has been included in subsection 4.2 (lines 701–714) of the revised manuscript.

**Q29**. References section apply the instructions to authors and use some recently published paper as a sample;

***Response:*** We have added some recently published papers as references [4,5, 38-41].

**Q30.** Write in red color the corrections you will do.

***Response:*** The manuscript has been revised, with all changes marked in red.

**Reviewer #2**

The study focuses on Nypa fruticans (Mangrove Palm) Fruit Kernel: A Novel Ingredient for Nutrient-Dense.
Low-Fat Biscuits. This study is very relevant and important since there is a need to use under fruits by products in the development of new food products. This is important since these fruit by products are rich in health promoting compounds. I have highlighted my comments and suggestions in the main document and the authors must address all of them, point by point. Below is the summary of the report:

**Q1**. Title, it needs to be revised to be in line with the content of the manuscript, i.e. *Nypa fruticans* (Mangrove Palm) Fruit Kernel: Effect on physicochemical, sensory evaluation and shelf-life of wheat biscuits.

***Response:*** Thank you. The requested task has been completed.

**Q2**. Abstract, should be straight to the point since it is a summary of results. Under color, the results of L*, a* and b* values should be clear rather saying darker colour.

***Response:*** The clarification has been added to the abstract (lines 28-30).

**Q3.** Introduction, it is sound, however, the knowledge gap and the novelty of the study is lacking. What is the current problem with wheat biscuits? How is the inclusion of Nypa fruticans fruit kernel addressing the problem? Previous studies done on the subject matter should be included to show what is lacking in the literature.

***Response:*** This has been completed (lines 88-98).

**Q4.** Materials and methods, it is very sound, the procedures for all the methods were explained in detail. However, section 2.2 should be summarized since the methods are known (AOAC standard methods).

***Response:*** Section 2.2 has been summarized.

**Q5.** Results and discussion, this section is very weak and must be strengthened to improve the scientific tone. There is a need to do statistical analysis for most of the tables. Otherwise, the discussion is meaningless if the data on the tables is speculated. The authors compared the data of raw flour and biscuit, but they did not indicate the sample used and why they chose the sample.

***Response:*** This has been discussed in lines 661–682.

Q6. This is important, authors must not speculate the biscuit sample, the information should be included under materials and methods. For color analyis, the discussion should be expanded by explaining the reason for increase in a* and decrease in b* values. The sensory evaluation section must be revised by doing statistical analysis of the results. The reason(s) should be explained in detail for sample with the highest value for each sensory attribute.

***Response:*** This has been discussed in lines 661–651.

**Q7.** Conclusion, should be in line with the objective of the study and should include practical implication of the obtained data.

***Response:*** Thank you. We have updated the conclusion.

**Q8.** References, I did not page numbers, but the style is consistent.

***Response:*** Thank you. We have added page number.

**Q9.** General comment, fruits by products are rich in phytochemicals, thus adding this section (total phenolic, total flavonoid, DPPH and FRAP) will improve the quality of the manuscript.

***Response:*** Added: Section 2.5 (225-265) and Section 3.4 (555-574).
