# Supplementary material for: Nypa fruticans wurmb (Mangrove Palm) Fruit Kernel: Effect on physicochemical, sensory evaluation and shelf-life of wheat biscuits
Source: PLoS One. 2026 Feb 18;21(2):e0342665. doi: 10.1371/journal.pone.0342665 (PMC12915934; doi:10.1371/journal.pone.0342665)
Supplement: Attachment — Submitted filename: Authors response to Reviewer Comments.docx [file pone.0342665.s003.docx]

The

Editor in Chief

PLOS ONE

Subject: Submission of review comments of the manuscript: “PONE-D-25-57201R1”

Dear Editor

I am submitting the review comments of the manuscript “*Nypa fruticans* Wurmb (Mangrove Palm) Fruit Kernel: Effect on physicochemical, sensory evaluation and shelf-life of wheat biscuits”. The authors would like to start by thanking the esteemed reviewers for their thoughtful comments and suggestions. A few points have been raised for clarification and further improvement of the paper. We have addressed each comment/suggestion made by the reviewers one by one below. We have also mentioned the changes made in the revised manuscript (m/s) below. All the changes in the revised m/s have been highlighted in Yellow and track change format.

**Journal Requirements:**

Q1. If the reviewer comments include a recommendation to cite specific previously published works, please review and evaluate these publications to determine whether they are relevant and should be cited. There is no requirement to cite these works unless the editor has indicated otherwise.

Response: Thank you for your query. Regarding your previous comments on the citations, we included references 3 and 4 because they are directly relevant to our work. Reference 3 examines the impact of glutamine-free conditions, while reference 4 reports on sensory and bioactivity testing, both of which support our study's framework.

2. Please review your reference list to ensure that it is complete and correct. If you have cited papers that have been retracted, please include the rationale for doing so in the manuscript text, or remove these references and replace them with relevant current references. Any changes to the reference list should be mentioned in the rebuttal letter that accompanies your revised manuscript. If you need to cite a retracted article, indicate the article’s retracted status in the References list and also include a citation and full reference for the retraction notice.

Response: Thank you for your query. We have checked **all references** and **added/retained only relevant and current ones.** Specifically, we have changed references **19–26 and 34**.

**Additional Editor Comments:**

Reviewer 1

Comment 3

In the title and at the first mention in the manuscript, when you write a scientific name, apply the International Botanical nomenclature and, after the species, insert the abbreviation of the Botanist (not italicised);

Response: The title has been corrected in accordance with your suggestion.

Academic Editor: Not satisfied.

Response: The title and at the first mention in the manuscript have been corrected in accordance with your suggestion.

Reviewer 1

Comment 7

Verify if to number the sections and the sub-sections;

Response: We have corrected the relevant section and subsection.

Academic Editor: Section 2.6 and section 4 appear twice. Please make these 2 changes and resubmit for final decision of your manuscript

Response: We have corrected the relevant section and subsection.
